# Age at onset as stratifier in idiopathic Parkinson’s disease – effect of ageing and polygenic risk score on clinical phenotypes

**DOI:** 10.1038/s41531-022-00342-7

**Published:** 2022-08-09

**Authors:** L. Pavelka, A. Rauschenberger, Z. Landoulsi, S. Pachchek, P. May, E. Glaab, R. Krüger, on behalf of the NCER-PD Consortium, Geeta Acharya, Gloria Aguayo, Myriam Alexandre, Muhammad Ali, Dominic Allen, Wim Ammerlann, Rudi Balling, Michele Bassis, Katy Beaumont, Regina Becker, Camille Bellora, Guy Berchem, Daniela Berg, Alexandre Bisdorff, Kathrin Brockmann, Jessica Calmes, Lorieza Castillo, Gessica Contesotto, Nico Diederich, Rene Dondelinger, Daniela Esteves, Guy Fagherazzi, Jean-Yves Ferrand, Manon Gantenbein, Thomas Gasser, Piotr Gawron, Soumyabrata Ghosh, Enrico Glaab, Clarissa Gomes, Elisa Gómez De Lope, Nikolai Goncharenko, Jérôme Graas, Mariella Graziano, Valentin Groues, Anne Grünewald, Wei Gu, Gaël Hammot, Anne-Marie Hanff, Linda Hansen, Maxime Hansen, Michael Heneka, Estelle Henry, Sylvia Herbrink, Eve Herenne, Sascha Herzinger, Michael Heymann, Michele Hu, Alexander Hundt, Nadine Jacoby, Jacek Jaroslaw Lebioda, Yohan Jaroz, Quentin Klopfenstein, Rejko Krüger, Pauline Lambert, Zied Landoulsi, Roseline Lentz, Inga Liepelt, Robert Liszka, Laura Longhino, Victoria Lorentz, Paula Cristina Lupu, Clare Mackay, Walter Maetzler, Katrin Marcus, Guilherme Marques, Tainá Marques, Patrick May, Deborah Mcintyre, Chouaib Mediouni, Francoise Meisch, Myriam Menster, Maura Minelli, Michel Mittelbronn, Brit Mollenhauer, Kathleen Mommaerts, Carlos Moreno, Serge Moudio, Friedrich Mühlschlegel, Romain Nati, Ulf Nehrbass, Sarah Nickels, Beatrice Nicolai, Jean-Paul Nicolay, Wolfgang Oertel, Marek Ostaszewski, Sinthuja Pachchek, Claire Pauly, Laure Pauly, Lukas Pavelka, Magali Perquin, Roslina Ramos Lima, Armin Rauschenberger, Rajesh Rawal, Dheeraj Reddy Bobbili, Eduardo Rosales, Isabel Rosety, Kirsten Rump, Estelle Sandt, Venkata Satagopam, Marc Schlesser, Margaux Schmitt, Sabine Schmitz, Reinhard Schneider, Jens Schwamborn, Amir Sharify, Ekaterina Soboleva, Kate Sokolowska, Olivier Terwindt, Hermann Thien, Elodie Thiry, Rebecca Ting Jiin Loo, Christophe Trefois, Johanna Trouet, Olena Tsurkalenko, Michel Vaillant, Mesele Valenti, Liliana Vilas Boas, Maharshi Vyas, Richard Wade-Martins, Paul Wilmes

**Affiliations:** 1 Clinical and Experimental Neuroscience, Luxembourg Centre for Systems Biomedicine (LCSB), University of Luxembourg, Esch-sur-Alzette, Luxembourg; 2 Parkinson’s Research Clinic, Centre Hospitalier de Luxembourg (CHL), Luxembourg, Luxembourg; 3 Biomedical Data Science Group, Luxembourg Centre for Systems Biomedicine (LCSB), University of Luxembourg, Esch-sur-Alzette, Luxembourg; 4 Bioinformatics Core, Luxembourg Centre for Systems Biomedicine (LCSB), Esch-sur-Alzette, Luxembourg; 5 Transversal Translational Medicine, Luxembourg Institute of Health (LIH), Strassen, Luxembourg; 6 Luxembourg Institute of Health, Strassen, Luxembourg; 7 Luxembourg Centre for Systems Biomedicine, University of Luxembourg, Esch-sur-Alzette, Luxembourg; 8 Centre Hospitalier de Luxembourg, Strassen, Luxembourg; 9 Center of Neurology and Hertie Institute for Clinical Brain Research, Department of Neurodegenerative Diseases, University Hospital Tübingen, Tübingen, Germany; 10 Centre Hospitalier Emile Mayrisch, Esch-sur-Alzette, Luxembourg; 11 Association of Physiotherapists in Parkinson’s Disease Europe, Esch-sur-Alzette, Luxembourg; 12 Centre Hospitalier du Nord, Ettelbrück, Luxembourg; 13 Oxford Parkinson’s Disease Centre, Nuffield Department of Clinical Neurosciences, University of Oxford, Oxford, UK; 14 Private practice, Ettelbruck, Luxembourg; 15 Parkinson Luxembourg Association, Leudelange, Luxembourg; 16 Westpfalz-Klinikum GmbH, Kaiserslautern, Germany; 17 Oxford Centre for Human Brain Activity, Wellcome Centre for Integrative Neuroimaging, Department of Psychiatry, University of Oxford, Oxford, UK; 18 Department of Neurology, University Medical Center Schleswig-Holstein, Kiel, Germany; 19 Ruhr-University of Bochum, Bochum, Germany; 20 Laboratoire National de Santé, Dudelange, Luxembourg; 21 Paracelsus-Elena-Klinik, Kassel, Germany; 22 Private practice, Luxembourg-City, Luxembourg; 23 Department of Neurology Philipps, University Marburg, Marburg, Germany; 24 Oxford Parkinson’s Disease Centre, Department of Physiology, Anatomy and Genetics, University of Oxford, Oxford, UK

**Keywords:** Parkinson's disease, Neurodegeneration, Dementia

## Abstract

Several phenotypic differences observed in Parkinson’s disease (PD) patients have been linked to age at onset (AAO). We endeavoured to find out whether these differences are due to the ageing process itself by using a combined dataset of idiopathic PD (*n* = 430) and healthy controls (HC; *n* = 556) excluding carriers of known PD-linked genetic mutations in both groups. We found several significant effects of AAO on motor and non-motor symptoms in PD, but when comparing the effects of age on these symptoms with HC (using age at assessment, AAA), only positive associations of AAA with burden of motor symptoms and cognitive impairment were significantly different between PD vs HC. Furthermore, we explored a potential effect of polygenic risk score (PRS) on clinical phenotype and identified a significant inverse correlation of AAO and PRS in PD. No significant association between PRS and severity of clinical symptoms was found. We conclude that the observed non-motor phenotypic differences in PD based on AAO are largely driven by the ageing process itself and not by a specific profile of neurodegeneration linked to AAO in the idiopathic PD patients.

## Introduction

Although considered as one disease entity, Parkinson’s disease (PD) displays substantial clinical heterogeneity with various phenotypes that translate into different combinations of both motor and non-motor symptoms. To address this heterogeneity, the age at onset (AAO) has been suggested as a key indicator associated with the clinical profile and progression of PD^1–3^. Previous studies with cross-sectional design have identified later AAO to be related with a stronger motor as well as non-motor impairment suggesting that late AAO is associated with higher progression rate of motor symptoms and cognitive decline. Conversely, early onset PD has been reported to show a specific disease profile with higher rate of motor complications such as early dyskinesia and dystonia^4–6^. Furthermore, both prospective^7^ and retrospective studies with autopsy-proven PD^8^ have shown similar findings, but given the heterogeneity of the study designs and various cut-offs used for categorising AAO, the reproducibility of the findings is limited. Despite reporting multiple AAO-related phenotypic differences, no study so far has endeavoured to integrate the effect of the physiological ageing process. Therefore, the associations between AAO and severity of PD phenotypes require further analysis.

Apart from AAO, the concept of polygenic risk scores (PRS) in sporadic forms of PD has recently been established to assess the complex genetic architecture of PD beyond known rare familial forms of PD with Mendelian inheritance of mutations in disease-causing genes^9^. Even though PRS were reported to be significantly negatively correlated with AAO^10^, potential effects of PRS on the disease severity and the phenotypic profile have not yet been explored in detail.

Previous studies focusing on the role of AAO in PD were limited by (i) not addressing the concomitant effect of the physiological ageing process on the clinical phenotype by modelling age-related effects in a healthy control group, (ii) including relatively small numbers of PD patients from highly specific subgroups (e.g. drug naïve), (iii) using different AAO cut-offs across the studies and (iv) lacking a detailed genetic profiling of the study sample to exclude individuals with monogenic forms and variants presenting a genetic risk factor for developing PD. Therefore, our study addresses these issues by combining a mono-centric idiopathic PD dataset and healthy control group (HC) with detailed genetic data with the aim (i) to investigate the effect of AAO on clinical phenotype in idiopathic PD, (ii) to separate the PD-related ageing effect from the natural ageing effect and finally (iii) to explore the effect of the genetic background reflected by PRS on the disease severity in idiopathic PD.

## Results

### Effect of AAO on clinical outcomes in PD

**Fig. 1:**
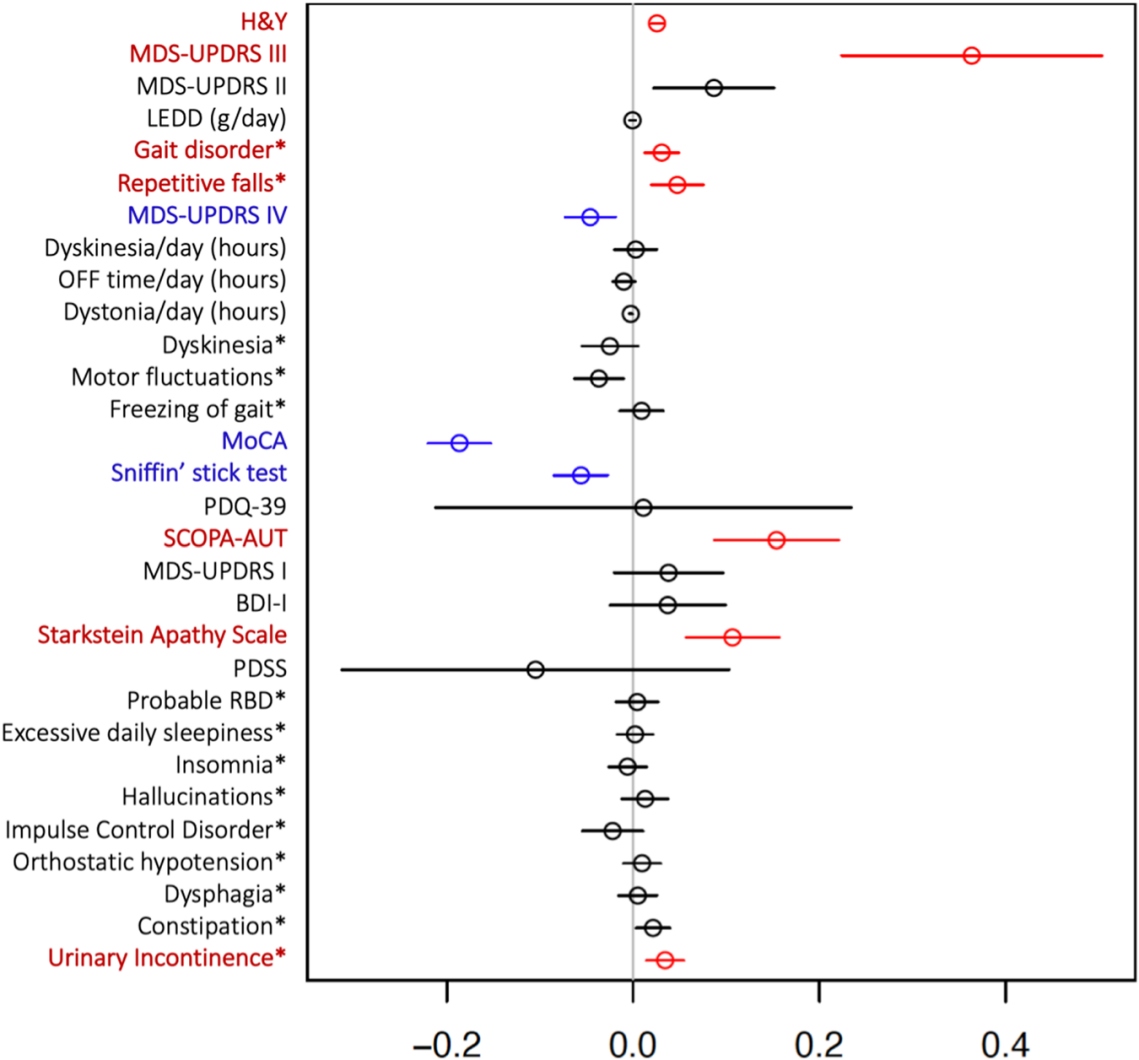
Forrest plot with estimated coefficients and corresponding confidence intervals (±1.96 × standard error) for AAO, from linear/logistic regression of numerical/binary outcome on disease duration and AAO. The colour blue indicates significant negative effects of AAO on the clinical outcome, and the colour red indicates significant positive effects at the Bonferroni-adjusted 5% level. The binary variables are annotated by asterisk. Clinical symptoms and scales are described in Supplementary Material.

Several traits in PD phenotypic profiles were found in association with AAO. An overview of clinical outcomes, sociodemographic characteristics and comorbidities among participants of the Luxembourg Parkinson’s Study is shown in Tables 1 and 2. As expected, the PD group comprised more males than females (67% vs. 33%) with mean AAO of 61.8 ± 12.0 years and mean disease duration since diagnosis of 5.5 ± 5.5 years. The mean age at assessment (AAA) was 67.3 ± 11.0 years. To investigate the effects of AAO on the clinical outcomes, a multiple regression analysis adjusting for disease duration was performed with results shown in Fig. 1. The overall motor disease severity as reflected by modified H&Y, MDS-UPDRS III, frequency of falls and gait disorder were all significantly positively associated with AAO. With regard to the motor complications of PD, no significant association of AAO was found with total hours of dyskinesia/day, dystonia/day, nor OFF time/day, however, a significant negative association of AAO with the MDS-UPDRS IV total score was identified. Additionally, SCOPA-AUT total score and Starkstein Apathy scale had significant positive associations with AAO indicating that patients with higher AAO experience more non-motor symptoms including urinary incontinence. Cognition as reflected by the MoCA score was significantly negatively associated with AAO showing higher impairment in patients with an older AAO. Similarly, AAO was significantly negatively associated with olfactory dysfunction. All other putative associations were not significantly associated with AAO as shown in Fig. 1.

**Table 1:** Overview of sociodemographic characteristics of study dataset including comorbidities and polygenic risk score with *p* values from Mann–Whitney *U* test for numerical variables and Fisher’s exact test for binary variables.

	HC *n* = 556			PD = 430			
Demographic, PRS and comorbidities	Mean or YES in %	SD or NO/YES	n.a.	Mean or YES in %	SD or NO/YES	n.a.	*p* value
Gender (male)*	56%	243/313	0	67%	142/288	0	7.8e−04″
Age at onset (years)	–	–	556	61.84	11.99	0	–
Age at assessment (years)	59.61	11.78	0	67.30	11.04	0	6.8e−23″
Disease duration since diagnosis (years)	–	–	556	5.49	5.54	0	–
Years of education	14.27	3.88	5	13.09	4.10	0	5.4e−06″
Family history of parkinsonism*	26%	408/146	2	25%	324/106	0	5.6e−01
Family history of dementia*	32%	373/178	5	24%	325/103	2	5.4e−03′
Polygenic risk score for PD	−0.21	0.91	6	0.16	0.94	6	7.1e−09″
De novo*	–	0/0	556	8%	395/35	0	–
Treatment with DBS*	0%	556/0	0	5%	410/20	0	4.8e−08″
History or presence of RLS*	6%	520/36	0	9%	392/38	0	1.8e−01
Diabetes (type not specified)*	6%	523/33	0	10%	385/45	0	1.2e−02′
Arterial hypertension*	33%	375/181	0	44%	239/191	0	1.6e−04″
Cardiovascular disease*	9%	504/52	0	21%	340/90	0	3.8e−07″
Hypercholesterolemia*	38%	347/209	0	42%	248/182	0	1.5e−01
History of stroke*	3%	539/17	0	5%	410/20	0	2.4e−01

Single and double ticks indicate significance at the 5% level and the Bonferroni-adjusted 5% level respectively. The binary variables are annotated by asterisk.

*n.a.* corresponds to total number of missing values per variable, *PD* Parkinson’s disease, *PRS* Polygenic risk score, *HC* Healthy controls, *DBS* Deep brain stimulation, *SD* Standard deviation.

**Table 2:** Overview of dataset with clinical variables in healthy control group (HC) and Parkinson’s disease patients (PD) with *p* values from Mann–Whitney *U* test for numerical variables and Fisher’s exact test for binary variables.

	HC *n* = 556			PD = 430			
Clinical symptoms and scales	Mean or YES in %	SD or NO/YES	n.a.	Mean or YES in %	SD or NO/YES	n.a.	*p* value
H&Y	0.00	0.00	2	2.24	0.81	2	1.5e−196″
MDS-UPDRS III	3.45	4.76	6	34.70	17.02	9	3.1e−150″
MDS-UPDRS II	1.21	2.37	6	11.69	8.32	8	6.2e−126″
LEDD (g/day)	0.0035	0.037	0	0.53	0.42	0	7.5e−160″
Gait disorder*	2%	546/10	0	57%	185/245	0	6.9e−97″
Repetitive falls*	1%	552/4	0	18%	351/79	0	1.1e−25″
MDS-UPDRS IV	0.00	0.00	4	1.88	3.52	5	1.4e−43″
Dyskinesia/day (hours)	0.00	0.00	0	0.69	2.73	1	1.2e−21″
OFF time/day (hours)	0.00	0.00	0	0.53	1.44	2	3.3e−34″
Dystonia/day (hours)	0.00	0.00	0	0.048	0.22	2	6.8e−12″
Dyskinesia*	0%	556/0	0	13%	375/55	0	1.9e−21″
Motor fluctuations*	0%	556/0	0	17%	357/73	0	1.1e−28″
Freezing of gait*	0%	556/0	0	23%	331/99	0	1.6e−39″
MoCA	27.03	2.55	3	24.28	4.41	8	4.4e−28″
Sniffin’ stick test	12.86	2.39	2	8.03	3.41	13	1.6e−94″
PDQ-39	10.31	13.20	16	39.69	26.31	39	1.3e−80″
SCOPA-AUT	7.34	5.81	16	14.82	8.02	21	6.9e−53″
MDS-UPDRS I	4.58	4.42	9	10.47	7.04	10	9.0e−51″
BDI-I	5.29	5.03	15	9.97	7.11	23	2.3e−30″
Starkstein Apathy Scale	9.41	4.71	16	13.93	5.70	26	8.2e−35″
PDSS	122.81	19.61	13	105.17	23.85	28	2.3e−34″
Probable RBD*	8%	496/42	18	24%	305/95	30	1.2e−11″
Excessive daily sleepiness*	3%	541/15	0	30%	299/131	0	2.4e−36″
Insomnia*	8%	514/42	0	24%	327/103	0	9.1e−13″
Hallucinations*	0%	554/2	0	17%	357/73	0	1.2e−25″
Impulse Control Disorder*	0%	555/1	0	9%	392/38	0	1.8e−13″
Orthostatic hypotension*	6%	525/31	0	27%	312/118	0	8.2e−22″
Dysphagia*	1%	552/4	0	25%	323/107	0	5.6e−37″
Constipation*	5%	528/28	0	42%	250/180	0	3.6e−47″
Urinary Incontinence*	5%	530/26	0	32%	293/137	0	3.6e−31″

Single and double ticks indicate significance at the 5% level and the Bonferroni-adjusted 5 % level respectively.

Clinical symptoms and scales are described in Supplementary Material. The binary variables are annotated by asterisk.

*n.a. (not acquired)* corresponds to total number of individuals with missing value, *SD* Standard deviation.

### Analysing the difference in ageing effect in PD vs HC

When investigating the effects of AAA and AAO on the clinical phenotypes of PD, all associations were found to be comparable in both models (cf. Table 3). The reason is the strong correlation between AAA and AAO (statistically significant Kendall’s tau ρ = 0.73, see Supplementary Fig. 1). To investigate an effect of physiological ageing on the PD phenotypes, we also included the HC group into the regression models. When investigating the ageing-associated effects in PD, we determined a significant positive association in PD between AAA and H&Y, MDS-UPDRS III, frequency of falls and urine incontinence, SCOPA-AUT, Starkstein Apathy Scale as well as significant negative association between AAA and MoCA and Sniffin’ Stick test (cf. Table 3). Similarly in the HC group, we found a significant positive association between AAA and MDS-UPDRS III, SCOPA-AUT, Starkstein Apathy Scale, frequency of urine incontinence and gait disorder as well as significant negative association between AAA and MoCA and Sniffin’ Stick test as demonstrated in Table 4. Surprisingly, after comparing the ageing effect between PD vs HC (i.e. comparing effect of AAA on the clinical variables; see Table 5, column AAA:status), the only significant differences between PD and HC were found for H&Y, MDS-UPDRS III, MDS-UPDRS IV and MoCA indicating that the concomitant ageing process might be the main determinant of the non-motor PD phenotypic differences when studying the isolated effect of age in PD.

**Table 3:** Multiple regression of clinical outcomes on age at onset (AAO), age at assessment (AAA) and disease duration for Parkinson’s disease group.

Clinical symptoms and scales	Intercept	Disease duration	AAA	Intercept	Disease duration	AAO	Intercept	AAA	AAO
H&Y	0.23	**0.05″**	**0.03″**	0.23	**0.07″**	**0.03″**	0.23	**0.07″**	**−0.05″**
MDS-UPDRS III	5.95	**0.76″**	**0.36″**	6.04	**1.13″**	**0.36″**	5.98	**1.13″**	**−0.76″**
MDS-UPDRS II	2.38	**0.63″**	0.09′	2.39	**0.72″**	0.09′	2.41	**0.72″**	**−0.63″**
LEDD (g/day)	0.37	**0.04″**	0.00	0.37	**0.03″**	0.00	0.38	**0.03″**	**−0.04″**
Gait disorder*	−2.23	**0.08″**	0.03′	−2.23	**0.12″**	0.03′	−2.22	**0.12″**	**−0.08″**
Repetitive falls*	−5.79	**0.14″**	**0.05″**	−5.74	**0.19″**	**0.05″**	−5.80	**0.19″**	**−0.15″**
MDS-UPDRS IV	3.43	**0.28″**	**−**0.05′	3.46	**0.24″**	−0.05′	3.43	**0.24″**	**−0.28″**
Dyskinesia/day (hours)	−0.16	**0.12″**	0.00	−0.16	**0.12″**	0.00	−0.15	**0.12″**	**−0.12″**
OFF time/day (hours)	0.87	**0.06″**	**−**0.01	0.87	**0.05″**	−0.01	0.87	**0.05″**	**−0.06″**
Dystonia/day (hours)	0.15	**0.01″**	0.00′	0.16	**0.01″**	0.00′	0.15	**0.01″**	**−0.01″**
Dyskinesia*	−1.57	**0.18″**	**−**0.02	−1.55	**0.15″**	−0.02	−1.56	**0.15″**	**−0.18″**
Motor fluctuations*	−0.35	**0.18″**	**−**0.04′	−0.39	**0.14″**	−0.04′	−0.33	**0.14″**	**−0.18″**
Freezing of gait*	−2.82	**0.16″**	0.01	−2.87	**0.17″**	0.01	−2.79	**0.17″**	**−0.16″**
MoCA	37.04	**−**0.05	**−0.19″**	37.10	**−0.24″**	**−0.19″**	37.03	**−0.23″**	**0.05**
Sniffin’ stick test	12.40	**−0.11″**	**−0.06″**	12.41	**−0.17″**	**−0.06″**	12.40	**−0.17″**	**0.11″**
PDQ-39	29.72	**1.78″**	0.01	29.46	**1.79″**	0.01	29.87	**1.77″**	**−1.76″**
SCOPA-AUT	2.20	**0.43″**	**0.15″**	2.16	**0.58″**	**0.15″**	2.23	**0.58″**	**−0.42″**
MDS-UPDRS I	6.05	**0.35″**	0.04	5.96	**0.39″**	0.04	6.08	**0.38″**	**−0.35″**
BDI-I	6.14	**0.24″**	0.04	6.19	**0.28″**	0.04	6.15	**0.28″**	**−0.24″**
Starkstein Apathy Scale	6.75	−0.01	**0.11″**	6.81	0.10	**0.11″**	6.74	0.10	0.00
PDSS	117.20	**−0.98″**	**−**0.10	117.45	**−1.08**″	−0.10	117.11	**−1.06″**	**0.96″**
Probable RBD*	−2.13	**0.11″**	0.00	−2.14	**0.12″**	0.00	−2.12	**0.11″**	**−0.11″**
Excessive daily sleepiness*	−1.31	**0.07″**	0.00	−1.37	**0.07″**	0.00	−1.29	0.07′	**−0.06″**
Insomnia*	−1.00	0.04′	**−**0.01	−1.01	0.04	−0.01	−1.00	0.04	**−0.04′**
Hallucinations*	−3.08	**0.09″**	0.01	−3.06	**0.10″**	0.01	−3.08	**0.11″**	**−0.09″**
Impulse Control Disorder*	−1.62	**0.11″**	**−**0.02	−1.64	0.09′	−0.02	−1.60	0.09′	**−0.11″**
Orthostatic hypotension*	−1.88	0.05′	0.01	−1.92	0.06′	0.01	−1.87	0.06′	−0.05′
Dysphagia*	−1.77	0.06′	0.00	−1.80	0.06′	0.01	−1.76	**0.06′**	−0.06′
Constipation*	−2.14	**0.07″**	0.02′	−2.19	**0.10″**	0.02′	−2.13	**0.09″**	**−0.07″**
Urinary Incontinence*	−3.40	0.04′	**0.04″**	−3.36	**0.08″**	**0.03″**	−3.41	**0.08″**	−0.05′

Regression coefficients for different outcomes (rows) from three equivalent models with each two out of three features (columns). Single and double ticks indicate significance at the 5% level and the Bonferroni-adjusted 5% level respectively. The bold indicates significant effect where minus value indicates negative significant effect and positive value positive significant effect respectively. The binary variables are annotated by asterisk. Clinical symptoms and scales are described in Supplementary Material.

**Table 4:** Simple regression of clinical outcomes with healthy controls.

Clinical symptoms and scales	Intercept	AAA
H&Y	0.00	0.00
MDS-UPDRS III	−3.70	**0.12″**
MDS-UPDRS II	−0.21	0.02′
LEDD (g/day)	−0.02	0.00′
Gait disorder*	−12.56	**0.13″**
Repetitive falls*	−5.14	0.00
MDS-UPDRS IV	0.00	0.00
Dyskinesia/day (hours)	0.00	0.00
OFF time/day (hours)	0.00	0.00
Dystonia/day (hours)	0.00	0.00
Dyskinesia*	−26.57	0.00
Motor fluctuations*	−26.57	0.00
Freezing of gait*	−26.57	0.00
MoCA	29.84	**−0.05″**
Sniffin’ stick test	15.44	**−0.04″**
PDQ-39	10.68	−0.01
SCOPA-AUT	2.53	**0.08″**
MDS-UPDRS I	3.12	0.02
BDI-I	4.06	0.02
Starkstein Apathy Scale	5.11	**0.07″**
PDSS	130.37	−0.13
Probable RBD*	−1.58	−0.02
Excessive daily sleepiness*	−6.53	0.05
Insomnia*	−2.00	−0.01
Hallucinations*	−3.31	−0.04
Impulse Control Disorder*	−4.32	−0.04
Orthostatic hypotension*	−3.92	0.02
Dysphagia*	−7.28	0.04
Constipation*	−2.37	−0.01
Urinary Incontinence*	−8.43	**0.08″**

Regression coefficients are shown from linear regression of numerical outcome and from logistic regression of binary outcome on age at assessment (AAA). Single and double ticks indicate significance at the 5% level and the Bonferroni adjusted 5% level, the bold indicates significant effect where minus value indicates negative significant effect and positive value positive significant effect respectively. The binary variables are annotated by asterisk. Clinical symptoms and scales are described in Supplementary Material.

**Table 5:** Multiple regression model with PD and HC investigating the difference in effect of ageing in HC (AAA) and in PD (AAA:status) adjusted for disease duration.

Clinical symptoms and scales	Intercept	AAA	Status	Disease duration	AAA:Status
H&Y	0.00	0.00	0.23	**0.05″**	**0.03″**
MDS-UPDRS III	−3.70	0.12′	9.65	**0.76″**	**0.24″**
MDS-UPDRS II	−0.21	0.02	2.59	**0.63″**	0.06′
LEDD (g/day)	−0.02	0.00	0.39	**0.04″**	0.00
Gait disorder*	−12.56	0.13′	10.34	**0.08″**	−0.10′
Repetitive falls*	−5.14	0.00	−0.66	**0.14″**	0.04
MDS-UPDRS IV	0.00	0.00	3.43	**0.28″**	**−0.05**″
Dyskinesia/day (hours)	0.00	0.00	−0.16	**0.12″**	0.00
OFF time/day (hours)	0.00	0.00	0.87	**0.06″**	−0.01
Dystonia/day (hours)	0.00	0.00	0.15	**0.01″**	0.00′
Dyskinesia*	−20.57	0.00	18.99	**0.18″**	−0.02
Motor fluctuations*	−20.57	0.00	20.22	**0.18″**	−0.04
Freezing of gait*	−20.57	0.00	17.75	**0.16″**	0.01
MoCA	29.84	**−0.05**″	7.20	−0.05	**−0.14**″
Sniffin’ stick test	15.44	**−0.04**″	−3.03	**−0.11**″	−0.01
PDQ-39	10.68	−0.01	19.04	**1.78″**	0.01
SCOPA-AUT	2.53	0.08′	−0.33	**0.43″**	0.07
MDS-UPDRS I	3.12	0.02	2.94	**0.35″**	0.01
BDI-I	4.06	0.02	2.09	**0.24″**	0.02
Starkstein Apathy Scale	5.11	**0.07″**	1.64	−0.01	0.04
PDSS	130.37	−0.13	−13.17	**−0.98**″	0.03
Probable RBD*	−1.58	−0.02	−0.54	**0.11″**	0.02
Excessive daily sleepiness*	−6.53	0.05	5.22	**0.07″**	−0.05
Insomnia*	−2.00	−0.01	0.99	0.04′	0.00
Hallucinations*	−3.31	−0.04	0.23	**0.09″**	0.05
Impulse Control Disorder*	−4.32	−0.04	2.70	**0.11″**	0.01
Orthostatic hypotension*	−3.92	0.02	2.04	0.05′	−0.01
Dysphagia*	−7.28	0.04	5.51	0.06′	−0.03
Constipation*	−2.37	−0.01	0.23	**0.07″**	0.03
Urinary Incontinence*	−8.43	**0.08″**	5.03	0.04′	−0.05′

Regression coefficients are shown for different outcomes (rows). Status takes the value 0 for HC and 1 for PD, the AAA:status is the interaction term of AAA and being PD (status = 1). Single and double ticks indicate significance at the 5% level and the Bonferroni-adjusted 5% level respectively, the bold indicates significant effect where minus value indicates negative significant effect and positive value positive significant effect respectively. The column AAA:status indicates whether the effect of AAA on clinical outcomes differs between PD and HC. The binary variables are annotated by asterisk. Clinical symptoms and scales are described in Supplementary Material.

### Correlation between AAO and PRS and its effect on severity of the PD phenotype

**Fig. 2:**
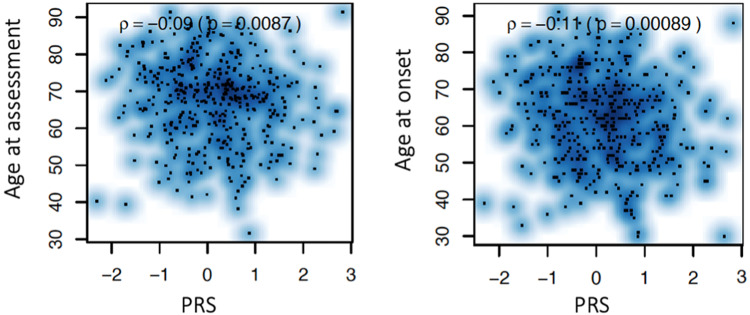
Pairwise association between age at onset (AAO), age at assessment (AAA) (y-axis) and polygenic risk score (PRS) (x-axis) with Kendall correlation coefficient. Significant inverse association was determined between AAO and PRS and AAA and PRS indicating the younger the AAO of PD, the higher cummulative burden of small effect size variants (represented by PRS).

Using a polygenic risk score defined by the imputed genotypic data from the Luxembourg Parkinson’s Study and the summary statistics of 90 single nucleotide polymorphisms (SNP) that were previously identified to be genome-wide significantly associated with PD risk, we identified a significant negative correlation between PRS and AAO as shown in Fig. 2. However, neither Kendall’s tau correlation test for continuous variables nor Mann–Whitney *U* test for binary variables estimating the effect of PRS on clinical outcomes nor multiple regression models including PRS adjusted for AAA and disease duration showed effects of PRS on the severity of the clinical phenotype as demonstrated in Tables 6 and 7 respectively.

**Table 6:** Kendall correlation coefficient between clinical outcome (row) and polygenic risk score (PRS) for healthy controls (HC) (left) and Parkinson’s disease patients (PD) (right), with annotation by bold indicating significant effect where minus value indicates negative significant effect and positive value positive significant effect respectively (Kendall correlation test).

Clinical symptoms and scales	HC	PD
H&Y	0.0272	0.0272
MDS-UPDRS III	−0.0088	−0.0341
MDS-UPDRS II	−0.0242	0.0058
LEDD (g/day)	0.0091	−0.0147
Gait disorder*	0.4070	−0.0177
Repetitive falls*	0.6209	0.1690
MDS-UPDRS IV	0.0625	0.0625
Dyskinesia/day (hours)	0.0576	0.0576
OFF time/day (hours)	0.0100	0.0100
Dystonia/day (hours)	0.0491	0.0491
Dyskinesia*	–	0.1426
Motor fluctuations*	–	0.0864
Freezing of gait*	–	0.0084
MoCA	0.0542	−0.0493′
Sniffin’ stick test	0.1012	0.0576
PDQ-39	−0.0519	0.0386
SCOPA-AUT	−0.0113	0.0150
MDS-UPDRS I	−0.0556	0.0105
BDI-I	−0.0155	0.0290
Starkstein Apathy Scale	−0.0425	−0.0068
PDSS	−0.0128	−0.0332
Probable RBD*	−0.1142	−0.0703
Excessive daily sleepiness*	0.1638	−0.1261
Insomnia*	−0.0356	0.1387
Hallucinations*	−0.9806	−0.1225
Impulse Control Disorder*	−2.1177	−0.0872
Orthostatic hypotension*	0.0839	−0.1331
Dysphagia*	−0.6401	−0.2650′
Constipation*	0.2701	−0.0740
Urinary Incontinence*	0.1011	0.0434

Single and double ticks indicate significance at the 5% level and the Bonferroni-adjusted 5% level. The binary variables are annotated by asterisk. Clinical symptoms and scales are described in Supplementary Material.

**Table 7:** Multiple regression model with coefficients shown from linear regression of numerical outcome and from logistic regression of binary outcome on disease duration, age at assessment (AAA) and polygenic risk score (PRS) in PD group.

Clinical symptoms and scales	Intercept	Disease duration	AAA	PRS
H&Y	0.17	**0.05″**	**0.03″**	−0.02
MDS-UPDRS III	4.44	**0.76″**	**0.39″**	−0.57
MDS-UPDRS II	1.81	**0.63″**	0.10′	−0.47
LEDD (g/day)	0.32	**0.04″**	0.00	0.01
Gait disorder*	−2.29	**0.09″**	0.03′	−0.04
Repetitive falls*	−5.91	**0.14″**	0.05′	0.15
MDS-UPDRS IV	3.37	**0.28″**	−0.04′	0.03
Dyskinesia/day (hours)	−0.36	**0.12″**	0.01	0.04
OFF time/day (hours)	0.87	**0.06″**	−0.01	−0.04
Dystonia/day (hours)	0.15	**0.01″**	0.00′	0.01
Dyskinesia*	−1.62	**0.18″**	−0.02	−0.05
Motor fluctuations*	−0.12	**0.18″**	−0.04′	−0.12
Freezing of gait*	−2.66	**0.17″**	0.01	−0.13
MoCA	37.25	−0.07′	**−0.19**″	0.40′
Sniffin’ stick test	12.64	**−0.11**″	**−0.06**″	−0.22
PDQ-39	30.96	**1.86″**	−0.01	−2.98′
SCOPA-AUT	2.74	**0.47″**	**0.15″**	−0.98′
MDS-UPDRS I	6.29	**0.36″**	0.03	−0.84′
BDI-I	6.81	**0.27″**	0.03	−0.76′
Starkstein Apathy Scale	6.99	0.00	**0.10″**	−0.18
PDSS	116.69	**−1.00**″	−0.10	0.12
Probable RBD*	−2.07	**0.12″**	0.00	−0.21
Excessive daily sleepiness*	−1.23	**0.07″**	0.00	−0.20
Insomnia*	−1.04	0.04′	−0.01	0.11
Hallucinations*	−2.95	**0.10″**	0.01	−0.23
Impulse Control Disorder*	−1.37	**0.11″**	−0.03	−0.25
Orthostatic hypotension*	−1.64	0.06′	0.01	−0.20
Dysphagia*	−1.77	**0.07″**	0.00	−0.37′
Constipation*	−2.07	**0.08″**	0.02′	−0.13
Urinary Incontinence*	−3.67	0.05′	**0.04″**	0.06

Single and double ticks indicate significance at the 5% level and the Bonferroni adjusted 5% level, the bold indicates significant effect where minus value indicates negative significant effect and positive value positive significant effect respectively. The binary variables are annotated by asterisk. Clinical symptoms and scales are described in Supplementary Material.

## Discussion

The presented cross-sectional analysis of PD patients and HC at the baseline clinical visit uses data from one of the largest ongoing observational studies, focusing on PD with demographic and clinical parameters corresponding closely to other recently published large PD datasets^11–13^. In our study, we have identified several significant associations of different PD-associated motor and non-motor symptoms with AAO using a comprehensive set of clinical assessments. This is in line with previous cross-sectional, retrospective and prospective studies suggesting that later onset PD is associated with a more rapid progression rate of motor symptoms^4,11,14,15^. Conversely, comparing to the Cardiff community-based PD longitudinal cohort^16^ and the longitudinal study at the Movement Disorders Clinic Saskatchewan^4^, both demonstrating higher frequency of dyskinesia, motor fluctuations and dystonia in the younger onset groups vs. older onset groups, we could not identify such associations with AAO. Only an overall burden of motor complications reflected by MDS-UPDRS IV score was significantly negatively associated with AAO in our study. The significant positive association of olfactory dysfunction and significant negative association of cognitive performance with AAO observed in our study correlate with previous findings^17,18^ and in terms of cognitive impairment it might point to a decreased ability of senescent brain to cope with the pathological neurodegenerative process known as cognitive resilience^19^. Additionally, another large multi-centric study using the Quebec Parkinson Network (QPN) dataset of over 1000 PD individuals showed comparable results with a positive association between late-onset PD and higher motor burden reflected by H&Y, higher cognitive decline and higher frequency of falls, but differed on significantly higher frequency of constipation and hallucinations late-onset PD (defined as AAO > 50 years) compared to early onset PD^11^. However, most scales applied in QPN differ from our study and different categorical approaches were used in QPN both for AAO and disease duration, influencing the comparability of results. To summarise our results, the earlier AAO, patients experience a lower level of motor impairment, lower cognitive impairment and less global autonomic dysfunction, apathy and olfactory deficit, but present with more motor complications even after adjusting for disease duration as a main determinant of disease severity.

These phenotypic differences observed in PD based on different AAO were previously not clearly separated from the physiological ageing process and challenged the concept that phenotypic differences are related specifically to the age at which the disease first manifests. This intriguing aspect evolves from the inherent close correlation between the main co-variates (AAA, AAO and disease duration) and thus raises a major methodological concern in most of the cross-sectional studies when aiming at determining the effect of all three co-variates on the clinical outcomes in a single model as discussed by Johnson et al. 2002^20^. Therefore, we tried to disentangle the effect of ageing on the clinical phenotype in the cross-sectional setting by determining the ageing effect in individuals with and without PD. Surprisingly, the effect of ageing (AAA) on clinical outcomes in PD vs HC differed significantly only in motor disease severity (H&Y, MDS-UPDRS III), motor complications (MDS-UPDRS IV) and cognitive performance. These results suggest that the majority of the observed significant non-motor phenotypic differences in PD should be attributed rather to the physiological ageing process itself than age-specific dynamics of PD.

When considering the effect and role of AAO and age in classification of the respective PD phenotypes, potential underlying genetic determinants need to be considered. It is well known that rare disease-causing mutations in monogenic PD (e.g. in PARKIN, PINK1, SNCA or GBA^21–23^) have an effect on both AAO and PD phenotype. However, until now only few studies have explored the cumulative effects of common genetic variants with small effect sizes (as defined by PRS) on the clinical phenotype^24^. Here our results are in line with several recent studies observing no significant association between PRS and cognitive decline, severity of motor symptoms^25^ or ICD^26^ in contrast to other longitudinal prospective study^27^. It is worth noting that our statistical models included individuals without any known PD causing monogenic mutation or genetic risk variant (i.e. PD-associated variants in the GBA gene). Nevertheless, the significance of the PRS effect on clinical outcomes did not change in the models including PD-associated mutation or genetic variant carriers. Together with the significant negative correlation between AAO and PRS (cf. Fig. 2), our findings suggest that PRS may increase the risk to develop PD but might not have an effect on the severity of the disease phenotype. This observation is in favour of the hypothesis that initiation of the disease on one hand and the disease progression rate on the other might be driven by distinct factors.

Besides the mentioned strengths of our study design, several limitations need to be considered. First, the cross-sectional design does not allow for the identification of causal relations between AAO and clinical phenotypes. Second, we cannot consider the Luxembourg Parkinson’s Study as community-based by design, although some clinical indicators (such as mean AAO and male-to-female ratio) correspond closely to several community-based studies^28–31^. Third, we observe a relatively high frequency of positive family history of parkinsonism in the HC group (26% vs. 25% in PD) as well as high frequency of a family history of dementia in HC (32% vs 24%). We assume that there are two principal reasons why we observe increased frequencies of neurodegenerative diseases in HC group: (i) HC with personal experience with parkinsonism and/or dementia in their family are more aware to support research and (ii) family members of study participants are more inclined to participate in the study. To address these points and eliminate a potential bias, we excluded 1^st^, 2^nd^ and 3^rd^ degree relatives from our statistical models.

In summary, our study sought to overcome limitations identified in previous studies on the role of AAO in PD by (i) including substantially higher number of PD patients and HCs in the model accounting for the independent effect of ageing, (ii) our study being based on monocentric data collection and including PD patients of all disease stages regardless of the cognitive status, (iii) investigating an idiopathic dataset of PD and PD-related mutation free HC, (iv) refuting the categorisation bias by a priori arbitrary AAO grouping, and finally (v) exploring the effect of PRS on severity of the PD phenotype in a large genotyped sample.

## Methods

### Study population

**Fig. 3:**
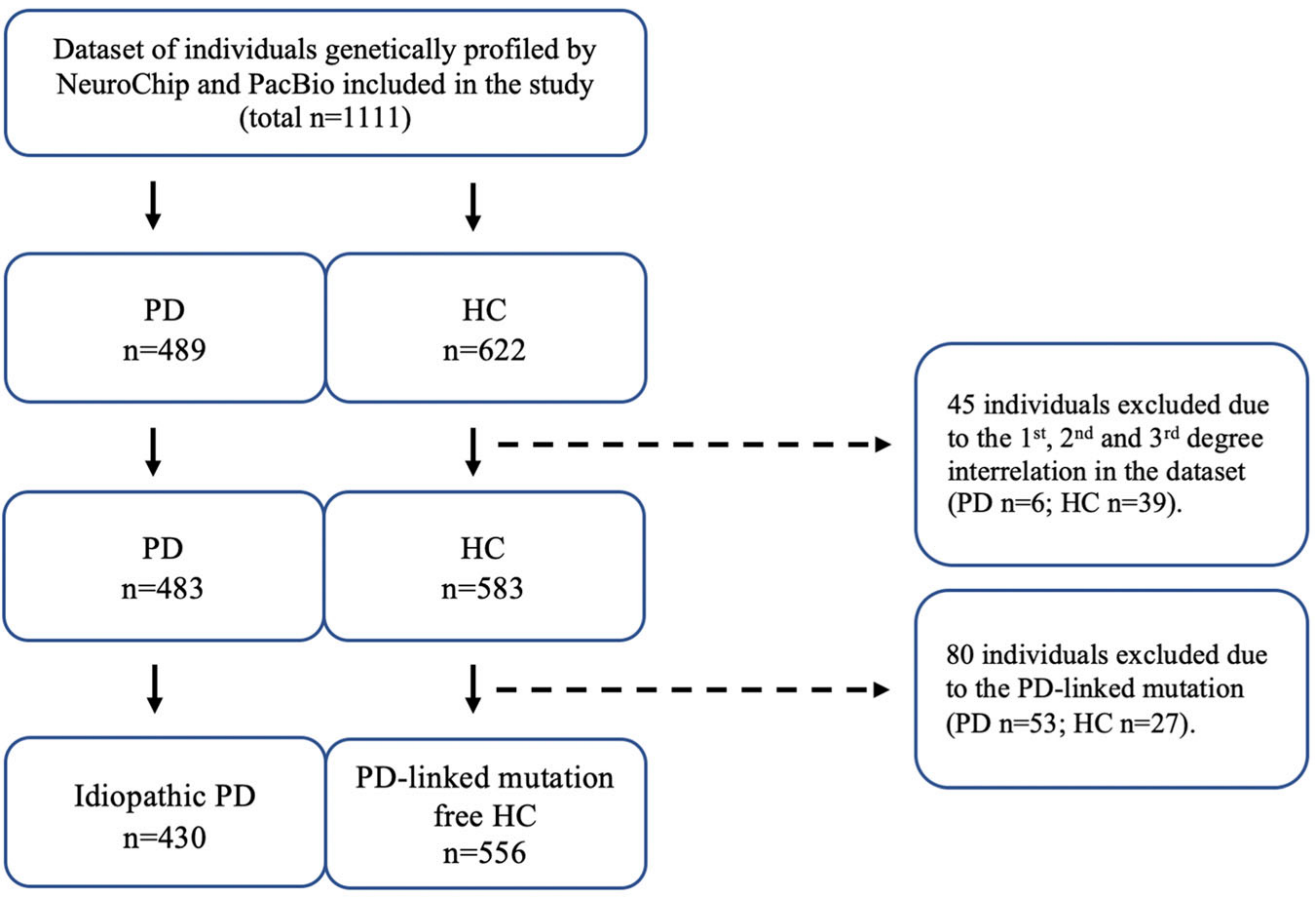
Description of the study design and study dataset. PD individuals with Parkinson’s disease, HC healthy control.

All subjects were recruited from March 2015 until 10th December 2020 in the frame of the nation-wide monocentric observational longitudinal Luxembourg Parkinson’s Study. The diagnosis of PD was based on UKPDSBB diagnostic criteria^32^. The initial visit dataset of 430 PD patients and 556 HC genetically screened by both NeuroChip and PacBio were analysed after exclusion of 6 PD and 39 HC individuals for 1^st^, 2^nd^ and 3^rd^ degree relationships and after exclusion of 53 PD carriers and 27 HC carriers of pathogenic PD-associated variants. The overall study design, inclusion and exclusion workflow are illustrated in Fig. 3.

All participants taking part in Luxembourg Parkinson’s Study agreed and signed a written informed consent. The study has been approved by the National Ethics Board (CNER Ref: 201407/13). The patients with PD were included regardless of the disease duration, cognitive status, age or disease stage. The HC were partially recruited from the pool of independent observational studies in Luxembourg (ORISCAV-LUX study; EHES-LUX) or were recruited from Luxembourg or the surrounding area of Greater Region based on individual interest not meeting any of the exclusion criteria (presence of a neurodegenerative disorder, active cancer; age under 18 and pregnant women)^33^.

#### Clinical assessment and data

A description of the design of the Luxembourg Parkinson’s Study was previously published^33^. Sociodemographic characteristics and clinical outcomes validated for PD were chosen from the basic clinical assessment battery and listed in Tables 1 and 2. Validated self-administered questionnaires and scales for PD were used. All patients have been evaluated in medication ON state and where applicable, in deep brain stimulation ON state. AAO is defined as age at diagnosis of PD. The clinical symptoms as scales are defined in detail in the Supplementary material.

#### Missing data statement

The absolute number of missing data per variable are shown in Tables 1 and 2. Given the low proportions of missing values in the outcome variables and 0% of missing values in the co-variates (AAA, AAO and disease duration), we used a pairwise deletion for all statistical models.

#### Genotyping and quality-control analyses

DNA samples were genotyped using the NeuroChip array (v.1.0 and v1.1; Illumina, San Diego, CA) that was specifically designed to integrate rare and common neurodegenerative disease-related variants^34^. Quality-control (QC) analysis was performed as follows: samples with call rates < 95% and whose genetically determined sex deviated from reported sex in clinical data were excluded from the analysis, and the filtered variants were checked for cryptic relatedness and excess of heterozygosity. Samples exhibiting excess heterozygosity (F statistic > 0.2) and first-degree relatedness were excluded. Once sample QC was completed, SNPs with Hardy−Weinberg equilibrium *P* value < 1E−6, and missingness rates >5% were excluded. All samples except for twelve from all individuals entering the analysis after exclusion of the 1^st^, 2^nd^ and 3^rd^ degree relatives and presence of PD-linked mutation and genetic risk factors passed the QC (424 PD and 550 HC). The data were then imputed using the Haplotype Reference Consortium r1.1 2016 and the Michigan Imputation Server and filtered for imputation quality (RSQ > 0.8)^35^. Genetic analysis and QC was done using PLINK v1.9. Additionally, all samples underwent targeted sequencing of the GBA locus using single-molecule sequencing on a Sequel II sequencer from Pacific BioScience^36^. Variants were called with DeepVariant 1.0^37^. PD causing rare variants were defined by the ClinVar classification ‘pathogenic/likely-pathogenic’. All PD causing variants (listed in Supplementary material) identified by any method were Sanger validated and all samples with a validated PD causing variant were excluded from further analysis.

#### Polygenic risk score (PRS)

We generated PRSs with PRSice-2 under default settings. PRSs for each individual were calculated using the imputed genotype data from Luxembourg Parkinson’s Study as a target sample. The base GWAS data used to determine PRS for PD was the summary statistics of the 90 SNPs that were previously found to be genome-wide significantly associated with PD risk^38^. The criteria for linkage disequilibrium (LD) clumping of SNPs were pairwise LD r2 < 0.1 within the 250 kb window. Briefly, PRSs were calculated by summing the weighted effects of GWAS PD risk genetic variants present in the target samples, with a possible proxy of R^2^ > 0.9, meeting *p* value thresholds ranging from 5e−08 to 0.5. The values of PRS were Z-normalised.

### Statistical analysis

Firstly, we performed an intergroup comparison (PD vs HC) of sociodemographic and clinical characteristics as well as polygenic risk score and comorbidities with the Mann−Whitney *U* test for numerical variables and Fisher’s exact test for binary variables (Tables 1 and 2). Secondly, we used multiple regression models (linear and logistic) to identify effects of AAO (as a numerical variable) on numerical or binary clinical outcomes accounting for disease duration (Fig. 1). Subsequently, we performed a multiple regression model for both HC and PD (Table 5) to examine whether the effect of ageing (AAA) on clinical outcomes differs between HC and PD adjusted for disease duration. For this, we included the main effects of the continuous variable AAA and the binary variable status (HC: status = 0, PD: status = 1), their interaction effect (HC: status*AAA = 0, PD: status*AAA > 0), and the main effect of the continuous variable disease duration (HC: duration = 0, PD: duration > 0). To investigate the role of PRS in PD, a pairwise association analysis with Kendall’s tau correlation test between PRS and AAO and AAA was performed (Fig. 2). Furthermore, we performed a Kendall correlation test between PRS and clinical outcome for PD and HC respectively (Table 6). As a last step, we employed a multiple regression model including PRS adjusting for AAA and disease duration, to investigate the effect of PRS on the clinical phenotype in PD (Table 7). At all instances, the significance at the 5% level and the Bonferroni-adjusted 5% level was set.

## Supplementary information

Supplementary material

## Data Availability

The dataset for this manuscript is not publicly available as it is linked to the Luxembourg Parkinson’s Study and its internal regulations. Any requests for accessing the dataset can be directed to *request.ncer-pd@uni.lu*.

## Code availability

The code for the statistical models is available at: 10.17881/hr67-ba06.
