# Publisher Correction: Age at onset as stratifier in idiopathic Parkinson’s disease – effect of ageing and polygenic risk score on clinical phenotypes

**DOI:** 10.1038/s41531-022-00378-9

**Published:** 2022-09-02

**Authors:** L. Pavelka, A. Rauschenberger, Z. Landoulsi, S. Pachchek, P. May, E. Glaab, R. Krüger, on behalf of the NCER-PD Consortium, Geeta Acharya, Gloria Aguayo, Myriam Alexandre, Muhammad Ali, Dominic Allen, Wim Ammerlann, Rudi Balling, Michele Bassis, Katy Beaumont, Regina Becker, Camille Bellora, Guy Berchem, Daniela Berg, Alexandre Bisdorff, Kathrin Brockmann, Jessica Calmes, Lorieza Castillo, Gessica Contesotto, Nico Diederich, Rene Dondelinger, Daniela Esteves, Guy Fagherazzi, Jean-Yves Ferrand, Manon Gantenbein, Thomas Gasser, Piotr Gawron, Soumyabrata Ghosh, Enrico Glaab, Clarissa Gomes, Elisa Gómez De Lope, Nikolai Goncharenko, Jérôme Graas, Mariella Graziano, Valentin Groues, Anne Grünewald, Wei Gu, Gaël Hammot, Anne-Marie Hanff, Linda Hansen, Maxime Hansen, Michael Heneka, Estelle Henry, Sylvia Herbrink, Eve Herenne, Sascha Herzinger, Michael Heymann, Michele Hu, Alexander Hundt, Nadine Jacoby, Jacek Jaroslaw Lebioda, Yohan Jaroz, Quentin Klopfenstein, Rejko Krüger, Pauline Lambert, Zied Landoulsi, Roseline Lentz, Inga Liepelt, Robert Liszka, Laura Longhino, Victoria Lorentz, Paula Cristina Lupu, Clare Mackay, Walter Maetzler, Katrin Marcus, Guilherme Marques, Tainá Marques, Patrick May, Deborah Mcintyre, Chouaib Mediouni, Francoise Meisch, Myriam Menster, Maura Minelli, Michel Mittelbronn, Brit Mollenhauer, Kathleen Mommaerts, Carlos Moreno, Serge Moudio, Friedrich Mühlschlegel, Romain Nati, Ulf Nehrbass, Sarah Nickels, Beatrice Nicolai, Jean-Paul Nicolay, Wolfgang Oertel, Marek Ostaszewski, Sinthuja Pachchek, Claire Pauly, Laure Pauly, Lukas Pavelka, Magali Perquin, Roslina Ramos Lima, Armin Rauschenberger, Rajesh Rawal, Dheeraj Reddy Bobbili, Eduardo Rosales, Isabel Rosety, Kirsten Rump, Estelle Sandt, Venkata Satagopam, Marc Schlesser, Margaux Schmitt, Sabine Schmitz, Reinhard Schneider, Jens Schwamborn, Amir Sharify, Ekaterina Soboleva, Kate Sokolowska, Olivier Terwindt, Hermann Thien, Elodie Thiry, Rebecca Ting Jiin Loo, Christophe Trefois, Johanna Trouet, Olena Tsurkalenko, Michel Vaillant, Mesele Valenti, Liliana Vilas Boas, Maharshi Vyas, Richard Wade-Martins, Paul Wilmes

**Affiliations:** 1 Clinical and Experimental Neuroscience, Luxembourg Centre for Systems Biomedicine (LCSB), University of Luxembourg, Esch-sur-Alzette, Luxembourg; 2 Parkinson’s Research Clinic, Centre Hospitalier de Luxembourg (CHL), Luxembourg, Luxembourg; 3 Biomedical Data Science Group, Luxembourg Centre for Systems Biomedicine (LCSB), University of Luxembourg, Esch-sur-Alzette, Luxembourg; 4 Bioinformatics Core, Luxembourg Centre for Systems Biomedicine (LCSB), Esch-sur-Alzette, Luxembourg; 5 Transversal Translational Medicine, Luxembourg Institute of Health (LIH), Strassen, Luxembourg; 6 Luxembourg Institute of Health, Strassen, Luxembourg; 7 Luxembourg Centre for Systems Biomedicine, University of Luxembourg, Esch-sur-Alzette, Luxembourg; 8 Centre Hospitalier de Luxembourg, Strassen, Luxembourg; 9 Center of Neurology and Hertie Institute for Clinical Brain Research, Department of Neurodegenerative Diseases, University Hospital Tübingen, Tübingen, Germany; 10 Centre Hospitalier Emile Mayrisch, Esch-sur-Alzette, Luxembourg; 11 Association of Physiotherapists in Parkinson’s Disease Europe, Esch-sur-Alzette, Luxembourg; 12 Centre Hospitalier du Nord, Ettelbrück, Luxembourg; 13 Oxford Parkinson’s Disease Centre, Nuffield Department of Clinical Neurosciences, University of Oxford, Oxford, UK; 14 Private practice, Ettelbruck, Luxembourg; 15 Parkinson Luxembourg Association, Leudelange, Luxembourg; 16 Westpfalz-Klinikum GmbH, Kaiserslautern, Germany; 17 Oxford Centre for Human Brain Activity, Wellcome Centre for Integrative Neuroimaging, Department of Psychiatry, University of Oxford, Oxford, UK; 18 Department of Neurology, University Medical Center Schleswig-Holstein, Kiel, Germany; 19 Ruhr-University of Bochum, Bochum, Germany; 20 Laboratoire National de Santé, Dudelange, Luxembourg; 21 Paracelsus-Elena-Klinik, Kassel, Germany; 22 Private practice, Luxembourg-City, Luxembourg; 23 Department of Neurology Philipps, University Marburg, Marburg, Germany; 24 Oxford Parkinson’s Disease Centre, Department of Physiology, Anatomy and Genetics, University of Oxford, Oxford, UK

**Keywords:** Parkinson's disease, Neurodegeneration, Dementia

Correction to: *npj Parkinson’s Disease* 10.1038/s41531-022-00342-7, published online 09 August 2022

In the legends for Tables 3, 4, 5, 6, and 7, the text “Bold indicates significant effects with negative values” should have read “The bold indicates significant effect where minus value indicates negative significant effect and positive value positive significant effect respectively”. This has now been corrected in both the PDF and HTML versions of the Article.

