# Supplementary material for: Age at onset as stratifier in idiopathic Parkinson’s disease – effect of ageing and polygenic risk score on clinical phenotypes
Source: NPJ Parkinsons Dis. 2022 Aug 9;8:102. doi: 10.1038/s41531-022-00342-7 (PMC9363416; doi:10.1038/s41531-022-00342-7)
Supplement: Supplementary file 1 — Supplementary material [file 41531_2022_342_MOESM1_ESM.pdf]

SUPPLEMENTARY MATERIAL

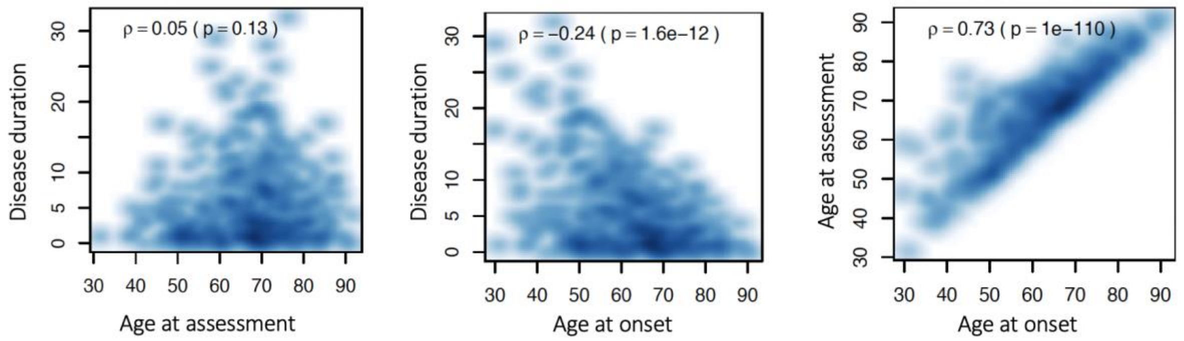

Supplementary Figure 1. Pairwise association among age at assessment (AAA), disease duration and age at onset (AAO) with Kendall correlation coefficient demonstrating a strong positive correlation between AAA and AAO.

Clinical symptoms and scales

Movement Disorder Society-Unified Parkinson's disease Rating Scale (MDS-UPDRS I-IV) and Scales for Outcomes in Parkinson's Disease-Autonomic questionnaire (SCOPA-AUT) are used under the license number (14017_ND). Clinical outcomes dystonia/day, OFF time/day were based on sub-items of MDS-UPDRS IV 4.6.2, 4.3.2. and 4.1.2. respectively. Probable REM sleep behaviour disorder (pRBD) was based on the validated self-reporting questionnaire REM Sleep Behaviour Disorder Screening Questionnaire (RBDSQ) where possible pRBD was defined as RBDSQ $\geq$ 7 [1]. Assessment of sleep quality was done via Parkinson's Disease Sleep Scale (PDSS) [2]. The calculation of levodopa equivalent daily dose (LEDD, reported in g/day) was based on established conversion factors [3]. *De novo* patient is defined as a dopaminergic drug naïve patient not more than one year since diagnosis of PD. Definition of constipation corresponds to the diagnostic criteria ROME III and information was acquired in a semi-structured interview [4]. The Hoehn and Yahr scale (H&Y) corresponds to the modified version of the scale [5]. Quality of life was assessed via Parkinson's disease Questionnaire 39 (PDQ-39) [6]. Depression symptoms were reflected by Beck Depression Inventory Version I (BDI-I) [7]. Olfactory function was examined with 16 items Sniffin' Stick test [8]. Cognitive performance was assessed via Montreal Cognitive Assessment (MoCA) [9]. Presence of recurrent orthostatic hypotension was assessed using a semi-structured interview inquiring for the symptoms of orthostatic hypotension, i.e. faintness, dizziness, light-headedness, vertigo, hearing disturbance, visual disturbance or syncope following the tilting, standing up or after a long standing relieved by sitting down or laying down. Symptoms included in the analysis (gait disorder, falls, freezing of gait (FOG), dyskinesia, motor fluctuations, excessive daily sleepiness, insomnia, dysphagia, urinary incontinence (corresponding to any type of urinary incontinence, i.e. stress, urge, overflow or mixed urinary incontinence), hallucinations and impulse control disorder (ICD)) were assessed during a semi-structured interview of the participant and/or the participant's proxy with a study physician and refer to the current motor and non-motor symptoms at the time of assessment.

List of genes and pathogenic PD causing variants excluded from the analysis

Heterozygote GBA p.N409S; heterozygote GBA p.E365K; one homozygote GBA p.E365K; GBA p.T408M; heterozygote GBA p.L444P; heterozygotes GBA p.R398X; GBA p.G241R; heterozygote GBA p.A215D; heterozygote GBA c.115+1G>A; heterozygote LRRK2 p.G2019S; homozygote PINK1 p.L369P; heterozygote GBA p.H294Q; heterozygote LRRK2 p.G2019S; dual heterozygote of LRRK2 p.R1441C and GBA p.E365K.

References to supplementary material

1. Takashi Nomura, Yuichi Inoue, Tatsuo Kagimura, Yusuke Uemura, Kenji Nakashima; Utility of the REM sleep behavior disorder screening questionnaire (RBDSQ) in Parkinson's disease patients, Sleep Medicine, Volume 12, Issue 7, 2011, Pages 711-713, ISSN 1389-9457, https://doi.org/10.1016/j.sleep.2011.01.015.
2. Chaudhuri KR, Pal S, DiMarco A, et al, The Parkinson's disease sleep scale: a new instrument for assessing sleep and nocturnal disability in Parkinson's disease, Journal of Neurology, Neurosurgery & Psychiatry 2002;73:629-635.
3. Tomlinson CL, Stowe R, Patel S, Rick C, Gray R, Clarke CE. Systematic review of levodopa dose equivalency reporting in Parkinson's disease. Mov Disord. 2010 Nov 15;25(15):2649-53. doi: 10.1002/mds.23429. PMID: 21069833.
4. Longstreth GF, Thompson WG, Chey WD, Houghton LA, Mearin F, Spiller RC. Functional bowel disorders. Gastroenterology. (2006) 130:1480– 91. doi: 10.1053/j.gastro.2005.11.061
5. Goetz CG, Poewe W, Rascol O, Sampaio C, Stebbins GT, Counsell C, Giladi N, Holloway RG, Moore CG, Wenning GK, Yahr MD, Seidl L; Movement Disorder Society Task Force on Rating Scales for Parkinson's Disease. Movement Disorder Society Task Force report on the Hoehn and Yahr staging scale: status and recommendations. Mov Disord. 2004 Sep;19(9):1020-8. doi: 10.1002/mds.20213. PMID: 15372591.
6. Peto V, Jenkinson C, Fitzpatrick R, Greenhall R. The development and validation of a short measure of functioning and well-being for individuals with Parkinson's disease. Qual Life Res. 1995 Jun;4(3):241-8. doi: 10.1007/BF02260863. PMID: 7613534.
7. Beck, A.T., Ward, C. H., Mendelson, M., Mock, J., & Erbaugh, J. (1961) An inventory for measuring depression. Archives of General Psychiatry, 4, 561-571.
8. Hummel T, Kobal G, Gudziol H, Mackay-Sim A. Normative data for the "Sniffin' Sticks" including tests of odor identification, odor discrimination, and olfactory thresholds: an upgrade based on a group of more than 3,000 subjects. Eur Arch Otorhinolaryngol 2007;264:237-243.
9. Nasreddine ZS, Phillips NA, Bédirian V, et al. The Montreal Cognitive Assessment, MoCA: a brief screening tool for mild cognitive impairment. J Am Geriatr Soc 2005;53:695–699
